# Comparison of longitudinal change in sST2 vs BNP to predict major adverse cardiovascular events in asymptomatic patients in the community

**DOI:** 10.1111/jcmm.15004

**Published:** 2020-04-29

**Authors:** Chris J. Watson, Isaac Tea, Eoin O'Connell, Nadezhda Glezeva, Shuaiwei Zhou, Stephanie James, Joe Gallagher, James Snider, James L. Januzzi, Mark T. Ledwidge, Ken M. McDonald

**Affiliations:** ^1^ Wellcome‐Wolfson Institute for Experimental Medicine Queen's University Belfast Belfast Northern Ireland; ^2^ STOP‐HF Group St Vincent's University Hospital Dublin Ireland; ^3^ UCD Conway Institute School of Medicine University College Dublin Dublin Ireland; ^4^ Internal Medicine Lankenau Medical Center Wynnewood PA USA; ^5^ Critical Diagnostics San Diego CA USA; ^6^ Cardiology Division Massachusetts General Hospital Boston MA USA

**Keywords:** biomarkers, BNP, cardiovascular event prediction, prognosis, ST2

## Abstract

Biomarker‐based preventative and monitoring strategies are increasingly used for risk stratification in cardiovascular (CV) disease. The aim of this study was to investigate the utility of longitudinal change in B‐type natriuretic peptide (BNP) and sST2 concentrations for predicting incident major adverse CV events (MACE) (heart failure, myocardial infarction, arrhythmia, stroke/transient ischaemic attack and CV death) in asymptomatic community‐based patients with risk factors but without prevalent MACE at enrolment. The study population consisted of 282 patients selected from the longitudinal STOP‐HF study of asymptomatic patients with risk factors for development of MACE. Fifty‐two of these patients developed a MACE. The study was run in two phases comprising of an initial investigative cohort (n = 195), and a subsequent 2:1 (No MACE: MACE) propensity matched verification cohort (n = 87). BNP and sST2 were quantified in all patients at two time points a median of 2.5 years apart. Results highlighted that longitudinal change in sST2 was a statistically significant predictor of incident MACE, (AUC 0.60). A one‐unit increment in sST2 change from baseline to follow up corresponded to approximately 7.99% increase in the rate of one or more incident MACE, independent of the baseline or follow‐up concentration. In contrast, longitudinal change value of BNP was not associated with MACE. In conclusion, longitudinal change in sST2 but not BNP was associated with incident MACE in asymptomatic, initially event‐free patients in the community. Further work is required to evaluate the clinical utility of change in sST2 in risk prediction and event monitoring in this setting.

## 1 INTRODUCTION

Current cardiovascular (CV) prevention strategies are becoming more focused on a biomarker approach to disease prediction, detection and monitoring, due to the potential economic benefits over imaging modalities, ease of use and ability to decentralize tests to community‐based settings, all of which lend to the option of frequent longitudinal testing and monitoring over time within large at‐risk populations.

Leading candidate biomarkers in the prevention setting include natriuretic peptide (NP) and soluble suppression of tumourigenicity 2 (sST2). Both biomarkers are related to CV stress and have been assessed in various CV disease settings, with value for prediction of risk for incident hospitalization, associated complications and death within heart failure populations.^1^, ^2^, ^3^ Application of these biomarkers in the asymptomatic setting to predict and/or track risk of future major adverse CV events (MACE) have also been investigated,^4^, ^5^, ^6^, ^7^, ^8^, ^9^ but most studies of this kind used a single time‐point measurement and change in sST2 in the prediction of future MACE within asymptomatic event‐free patients is yet to be investigated. Thus, it is not known if changes in biomarker levels over time in at‐risk populations have value in the prediction of future MACE. Biomarker change over time monitoring will have added strengths than single time‐point measurements as this approach could more accurately track new onset of disease in large at‐risk populations, and may also be applied in tracking response to interventions. Therefore, the aim of this study was to investigate longitudinal change in NP and sST2 biomarker levels over time in asymptomatic event‐free populations and assess their ability to predict future MACE.

## 2 METHODS

### 2.1 Study population

The study population consisted of 282 patients from within the STOP‐HF cohort,^5^ an ongoing prospective longitudinal study population in Ireland who have CV risk factors for the future development of MACE. Further details in Appendix S1. All study participants gave written informed consent to join the STOP‐HF cohort. The study protocol was approved by the ethics committee of St. Vincent's University Hospital, Dublin, which conformed to the principles of the Helsinki Declaration.

For the purpose of this study, two independent sub‐populations were selected from within the STOP‐HF cohort to investigate biomarker change over time and their ability to predict new onset MACE. Study population 1 consisted of 195 patients, subdivided into 23 patients who developed incident MACE and 172 patients who did not develop a MACE over a similar time period. The verification study population 2 consisted of 87 patients, propensity matched on age and sex at a ratio of 2:1 for no MACE and incident MACE. MACE identification and classification is described in Appendix S1. Of cautionary note, study population 2 is not a diagnostic validation cohort as the population characteristics do not match study population 1.

### 2.2 Biomarker measurements

Serum levels of sST2 were quantified using the Presage ST2 Assay (Critical Diagnostics) using frozen samples. Point‐of‐care B‐type natriuretic peptide (BNP) was measured at each visit (Triage, Biosite). Assay performance characteristics detailed within Appendix S1.

## 3 RESULTS

### 3.1 Patient characteristics of study population

The baseline description of the patient populations are highlighted in Table 1. Age, sex and co‐morbidities were similar in both study cohorts when comparing no MACE with MACE groups.

**Table 1:** Comparison of MACE and non‐MACE baseline results in pooled study and Study 1 & 2

Median [IQR]/n (%)	All			Study 1			Study 2		
	No MACE	MACE	*P*	No MACE	MACE	*P*	No MACE	MACE	*P*
	(n = 230)	(n = 52)		(n = 172)	(n = 23)		(n = 58)	(n = 29)	
Age	67.7 [61.5:74.3]	64.8 [60.7:69.9]	.025^W^	70.6 [65.1:75.3]	66.5 [63.6:72.7]	.258^W^	61.0 [58.1:67.7]	61.3 [58.2:67.7]	.857^W^
Male	126 (54.8%)	28 (53.8%)	>.99^c^	96 (55.8%)	13 (56.5%)	>.99^c^	30 (51.7%)	15 (51.7%)	>.99^c^
Diabetes mellitus	37 (16.1%)	14 (26.9%)	.102^c^	31 (18.0%)	7 (30.4%)	.258^c^	6 (10.3%)	7 (24.1%)	.167^c^
Hypertension	168 (73.0%)	42 (80.8%)	.328^c^	124 (72.1%)	19 (82.6%)	.412^c^	44 (75.9%)	23 (79.3%)	.928^c^
Hypercholesterolemia	176 (76.5%)	41 (78.8%)	.859^c^	130 (75.6%)	15 (65.2%)	.415^c^	46 (79.3%)	26 (89.7%)	.366^c^
Atrial fibrillation	13 (5.7%)	3 (5.8%)	>.99^f^	9 (5.2%)	1 (4.3%)	>.99^f^	4 (6.9%)	2 (6.9%)	>.99^f^
Myocardial Infarction	22 (9.6%)	12 (23.1%)	.014^c^	18 (10.5%)	6 (26.1%)	.071^c^	4 (6.9%)	6 (20.7%)	.077^f^
Stroke/TIA	7 (3.0%)	2 (3.8%)	>.99^f^	5 (2.9%)	2 (8.7%)	.194^f^	2 (3.4%)	0 (0.0%)	‐
Cancer	9 (3.9%)	4 (7.7%)	.268^f^	8 (4.7%)	3 (13.0%)	.126^f^	1 (1.7%)	1 (3.4%)	>.99^f^
COPD	4 (1.7%)	3 (5.8%)	.12^f^	2 (1.2%)	2 (8.7%)	.069^f^	2 (3.4%)	1 (3.4%)	>.99^f^
Baseline sST2	27.5 [21.4:35.4]	28.9 [23.7:34.8]	.237^W^	25.4 [20.0:33.4]	23.7 [21.4:26.7]	.473^W^	32.9 [28.1:41.5]	32.8 [29.1:39.0]	.882^W^
Follow‐up sST2	28.1 [22.3:35.1]	32.1 [26.4:38.3]	.007^W^	26.5 [20.3:32.5]	26.8 [23.6:32.9]	.457^W^	31.5 [27.4:40.5]	35.5 [30.5:39.2]	.24^W^
sST2 change	0.43 [−2.2:3.0]	2.0 [−0.65:4.5]	.016^W^	0.76 [−2.3:3.9]	2.7 [−1.7:8.8]	.049^W^	‐0.49 [−1.7:1.3]	1.3 [−0.37:3.8]	.053^t^
Baseline BNP	25.0 [15.0:54.0]	34.8 [18.5:88.9]	.036^W^	27.0 [15.7:59.6]	59.0 [23.3:138]	.018^W^	21.0 [10.2:43.8]	25.1 [17.5:49.0]	.131^W^
Follow‐up BNP	33.4 [15.5:70.6]	45.9 [15.8:107]	.061^W^	37.9 [17.4:73.0]	83.3 [24.9:178]	.014^W^	21.9 [12.0:52.9]	35.2 [14.1:73.9]	.224^W^
BNP change	3.4 [−7.1:23.8]	1.0 [−9.1:27.9]	.733^W^	4.2 [−5.6:24.9]	0.40 [−24.4:27.9]	.449^W^	1.7 [−8.0:16.7]	5.3 [−6.0:24.8]	.729^W^
Baseline EF	66 [60:73]	66 [60:72]	.487^W^	66 [60:72]	66 [61:73]	.678^W^	67 [62:74]	65 [59:71]	.064^W^

Abbreviations: BNP, b‐type natriuretic peptide; *c*, chi‐squared test; COPD, chronic obstructive pulmonary disease; EF, (left ventricular) ejection fraction; *f*, Fisher's exact test; IQR, interquartile range; MACE, major adverse cardiovascular event; sST2, soluble suppression of tumourigenicity 2; *t*, Student's t test; TIA, transient ischaemic attack; W, Wilcoxon signed‐rank test.

### 3.2 sST2 and BNP as predictors of MACE

A significant difference in sST2 longitudinal change was detected between the MACE and no MACE group in both study cohorts (*P* ≤ .05). BNP concentrations at baseline and follow‐up differed between the MACE and no MACE groups but only in Study 1 (*P* ≤ .05). BNP change and sST2 concentrations (at baseline and follow‐up) did not differ between the MACE and no MACE groups in either study sample. See Table 1 for details.

### 3.3 Cox proportional hazards modelling

The Kaplan‐Meier curves landmarked after follow‐up are shown in Figure S1. The average time to MACE event for all participants, Study 1 and Study 2 are 303, 216 and 372 days, respectively.

The summary of eight covariate variables, that is age, gender, sST2 at baseline, follow‐up and change, log‐transformed BNP at baseline, follow‐up and change, in the univariate analysis for Study 1 and Study 2, is exhibited in, Table S1 and Table S2. It can be seen that only sST2 change and log‐transformed BNP at baseline presented significance in univariate analysis in both studies. Multivariate analysis (Table S3 and Table S4) indicates that the model is not improved by adding these covariates (except with age in Study 1).

The results of the fitted model with sST2 change and log‐transformed BNP baseline as the predictors in Table S5 are interpreted in terms of hazard ratios (HR) for Study 1 and Study 2. sST2 change is the only significant predictor across both study cohorts. Being weighted by number of participants in each study, the general hazard ratio is 1.0774, that is, the patients with sST2 increase in 1 ng/mL have about 7.99% higher MACE risk than those who do not increase.

Area under curve (AUC) for sST2 and BNP at baseline, follow‐up, longitudinal change and change percentage and incident MACE is presented, see Table S6 and Table S7. The ROC properties of sST2 change were also presented in Table S8 via different threshold criteria, that is the arbitrarily optimal criterion as above, high sensitivity and high specificity.

**Figure 1:**
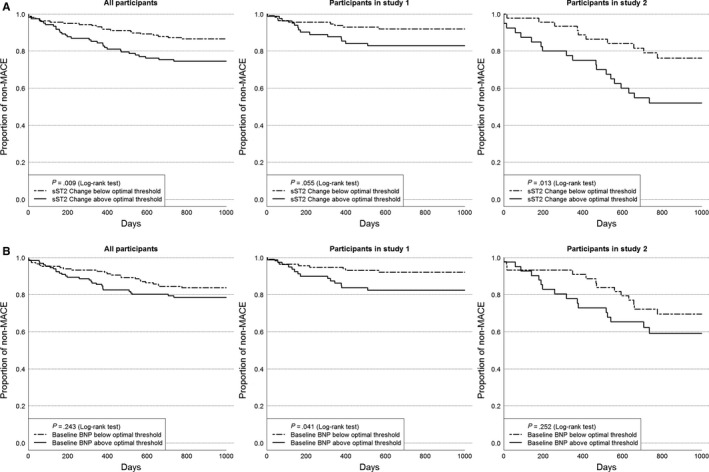
A, Kaplan‐Meier survival curves with sST2 change above and below the optimal thresholds from corresponding ROCs for all participants, Study 1 and Study 2. B, Kaplan‐Meier survival curves with baseline BNP above and below the optimal thresholds from corresponding ROCs for all participants, Study 1 and Study 2

Generally, a one‐unit increment in sST2 change corresponded to approximately 7.99% increase in the rate of one or more MACE. Utilizing the optimal thresholds, which were 14.7 ng/mL, 15.6 ng/mL and 14.2 ng/mL for all participants, Study 1 and Study 2 Kaplan‐Meier curves demonstrating time to first MACE event reveal participants with significant sST2 rise have higher proportion of experiencing MACE (Figure 1A). A similar analysis using baseline BNP optimal thresholds (All: 28.4 pg/mL, Study 1:36.1 pg/mL, Study 2:22.1 pg/mL) indicated no relationship between this binary grouping and MACE, except in Study 1, where there was a positive association (Figure 1B).

## 4 DISCUSSION

Our study reports longitudinal change in sST2 is a statistically significant predictor of MACE in an asymptomatic, event‐free, ambulatory population with cardiovascular risk factors. In this population, we found a one‐unit increment in sST2 corresponded to approximately 7.99% increase in the rate of one or more incident MACE independent of the baseline or follow‐up values; a patient with an sST2 longitudinal change of 20 ng/mL to 23 ng/mL has the same expected increase in the rate of having a MACE as one whose sST2 changes from 35 ng/mL to 38 ng/mL. In both study cohorts, change in sST2 was superior to BNP in predicting MACE. BNP was only significant in predicting MACE in the initial investigative cohort, and only with baseline and follow‐up BNP values. Longitudinal change in sST2, on the other hand was significant as a predictor of MACE in both of the study cohorts. This finding is further strengthened by the fact that inclusion of baseline or follow‐up BNP as covariates with change in sST2 did not improve either study cohort, while in contrast longitudinal change in sST2 in the three BNP models (baseline, follow‐up, change) significantly improved all models. These findings suggest longitudinal change in sST2 is a statistically significant predictor of MACE prevalence in this population of Irish adults with one or more CV risk factors.

In our study, the prognostic information offered by serial sST2 measurements appears additive over BNP. These results within an asymptomatic population correlate well with those of other investigators who also found that serial sST2 monitoring in the setting of established heart failure was superior to natriuretic peptides (BNP and NT‐proBNP) and are not affected by renal function or other confounders (age, sex and BMI) that often affect biomarker accuracy.^10^, ^11^, ^12^, ^13^, ^14^, ^15^ Their data, like ours, provide supportive evidence of the dynamic nature of sST2 values and the potential importance of serial sST2 measurements as a tool to guide the aggressiveness of therapy required for patients with (or at risk for) HF. However, further longitudinal studies within asymptomatic populations would be required in an attempt to better refine and improve the prognostic value of serial sST2, perhaps in conjunction with additional biomarkers.

## CONFLICTS OF INTEREST

Dr Januzzi has received grant support from Siemens, Singulex and Prevencio, consulting income from Roche Diagnostics, Critical Diagnostics, Sphingotec, Phillips, & Novartis, & participates in clinical end‐point committees/data safety monitoring boards for Novartis, Amgen, Janssen, & Boehringer Ingelheim. Dr James Snider works for Critical Diagnostics, the provider of the sST2 assay. Professor McDonald receives consulting income from Novartis, Ireland & Cruinn Diagnostics.

## AUTHOR CONTRIBUTION

CW designed the research study, performed the research and wrote the paper; IT performed the research and wrote the paper; EO, SZ analysed the data; NG, SJ and JG performed the research; JS and JL designed the research study; ML and KM designed the research study and wrote the paper.

## Data Availability

The data that support the findings of this study are not publicly available due to ethical restrictions.
