# Supplementary material for: Comparison of longitudinal change in sST2 vs BNP to predict major adverse cardiovascular events in asymptomatic patients in the community
Source: J Cell Mol Med. 2020 Apr 29;24(11):6495–9. doi: 10.1111/jcmm.15004 (PMC7294165; doi:10.1111/jcmm.15004)
Supplement: Supplementary file 1 [file JCMM-24-6495-s001.docx]

**Supplementary FILE**

**Appendix S1**

**Clinical measurements and biomarker assay characteristics**

All study participants gave written informed consent to join the STOP-HF cohort. The study protocol was approved by the ethics committee of St. Vincent’s University Hospital, Dublin, which conformed to the principles of the Helsinki Declaration.

At clinical visits, a full physical examination, New York Heart Association (NYHA) functional class assessment, and phlebotomy were performed by a blinded observer. Physical examination included assessment of waist circumference, body-mass index (BMI) calculation, heart rate and blood pressure measurement.

Assay sensitivity for quantification of sST2 and BNP was 2.35 ng/mL and 5 ng/mL, respectively. All subjects had detectable levels of sST2 at baseline and follow-up, whereas the lowest limit of detection for BNP (≤5ng/mL) was reported in 5% of subjects at baseline and 4% at follow-up.

**MACE identification and classification**

The STOP-HF population had cardiovascular risk factors for the future development of MACE, including heart failure, myocardial infarction, arrhythmia, stroke, transient ischemic attack, and CV death. Patients recruited are aged ≥ 40 years and have at least one risk factor for left ventricular dysfunction including hypertension, type 2 diabetes mellitus (DM), obesity, coronary artery disease, or hypercholesterolemia.

MACE was defined as an emergency hospitalization for any of the following major adverse cardiovascular events: arrhythmia, transient ischemic attack, stroke, myocardial infarction, peripheral or pulmonary thrombosis/embolus, heart failure, or CV death ^(PMID: 23821090)^. All MACE events were classified based on the discharge summary from the hospitalization event, and the investigators did not have any influence in the decision to admit. All clinical end points were assessed by a member of the study team by reviewing the primary care physician’s records and confirmed by hospital discharge summary. This process was carried out by the same specialist nurse for consistency of reporting over the study duration.

**Follow up durations**

The average, minimum and maximum follow up after the baseline are 1080, 177 and 2465 days. The average, minimum and maximum duration from follow up until the end of studies are 1259, 0 and 2892 days; 25% of the participants had 1520 days of follow up (upper quartile).

**Supplementary Results**

**Supplementary Tables**

**Table S1.** Results of the univariate proportional hazards Cox regression model of patients in Study 1 within the longitudinal study

| Predictor | Wald p-value | HR | 95% CI |
| --- | --- | --- | --- |
| Age | 0.58 | 0.99 | 0.94 - 1.03 |
| Gender | 0.93 | 1.04 | 0.46 - 2.37 |
| logBNP Baseline | 0.006 | 1.76 | 1.18 - 2.65 |
| logBNP Follow up | 0.007 | 1.81 | 1.17 - 2.79 |
| logBNP Change | 0.85 | 1.05 | 0.60 - 1.84 |
| sST2 Baseline | 0.26 | 0.98 | 0.93 - 1.02 |
| sST2 Follow up | 0.46 | 1.01 | 0.98 - 1.04 |
| sST2 Change | <0.001 | 1.07 | 1.03 - 1.11 |
| EF Baseline | 0.84 | 1.00 | 0.97 - 1.05 |

HR-hazard ratio; BNP – b-type natriuretic peptide; sST2 – soluble suppression of tumourigenicity 2, EF – (left ventricular) ejection fraction.

**Table S2.** Results of the univariate proportional hazards Cox regression model of patients in Study 2 within the longitudinal study

| Predictor | Wald p-value | HR | 95% CI |
| --- | --- | --- | --- |
| Age | 0.74 | 1.01 | 0.96 - 1.06 |
| Gender | 0.77 | 1.12 | 0.54 - 2.31 |
| logBNP Baseline | 0.034 | 1.54 | 1.03 - 2.28 |
| logBNP Follow up | 0.10 | 1.35 | 0.94 - 1.93 |
| logBNP Change | 0.67 | 0.89 | 0.52 - 1.51 |
| sST2 Baseline | 0.67 | 0.99 | 0.95 - 1.03 |
| sST2 Follow up | 0.67 | 1.01 | 0.97 - 1.05 |
| sST2 Change | 0.016 | 1.14 | 1.03 - 1.27 |
| EF Baseline | 0.046 | 0.97 | 0.94 - 0.99 |

HR-hazard ratio; BNP – b-type natriuretic peptide; sST2 – soluble suppression of tumourigenicity 2, EF – (left ventricular) ejection fraction.

**Table S3.** Multivariate models in Study 1. Adding single variables to the base model of variables significant in overall univariate analyses, logBNP baseline and sST2 change.

| Variables | LL($\hat{\beta}$) | -2LL($\hat{\beta}$) difference ($\chi^{2}$) | $\chi^{2}$ p-value |
| --- | --- | --- | --- |
| sST2 change + logBNP Baseline | -112.81 | - | - |
| sST2 change + logBNP Baseline + Age | -110.71 | 4.21 | 0.04 |
| sST2 change + logBNP Baseline + Gender | -112.77 | 0.08 | 0.78 |
| sST2 change + logBNP Baseline + sST Baseline | -112.12 | 1.39 | 0.24 |
| sST2 change + logBNP Baseline + sST Follow up | -112.12 | 1.39 | 0.24 |
| sST2 change + logBNP Baseline + logBNP Follow up | -112.40 | 0.82 | 0.37 |
| sST2 change + logBNP Baseline + logBNP Change | -112.40 | 0.82 | 0.37 |
| sST2 change + logBNP Baseline + EF Baseline | -112.74 | 0.14 | 0.71 |

sST2 – soluble suppression of tumourigenicity 2; BNP – b-type natriuretic peptide; EF – (left ventricular) ejection fraction

**Table S4.** Multivariate models in Study 2. Adding single variables to the base model of variables significant in overall univariate analyses, logBNP baseline and sST2 change.

| Variables | LL($\hat{\beta}$) | -2LL($\hat{\beta}$) difference ($\chi^{2}$) | $\chi^{2}$ p-value |
| --- | --- | --- | --- |
| sST2 change + logBNP Baseline | -117.75 | - | - |
| sST2 change + logBNP Baseline + Age | -117.50 | 0.51 | 0.48 |
| sST2 change + logBNP Baseline + Gender | -117.75 | 0.01 | 0.91 |
| sST2 change + logBNP Baseline + sST Baseline | -117.66 | 0.20 | 0.66 |
| sST2 change + logBNP Baseline + sST Follow up | -117.66 | 0.20 | 0.66 |
| sST2 change + logBNP Baseline + logBNP Follow up | -117.65 | 0.21 | 0.64 |
| sST2 change + logBNP Baseline + logBNP Change | -117.65 | 0.21 | 0.64 |
| sST2 change + logBNP Baseline + EF Baseline | -116.67 | 2.16 | 0.14 |

sST2 – soluble suppression of tumourigenicity 2; BNP – b-type natriuretic peptide; EF – (left ventricular) ejection fraction

**Table S5.** Cox proportional hazards modelling via sST2 change and logBNP baseline predicting MACE

| Analysis of maximum likelihood estimates | | | | | | | | | | | |
| --- | --- | --- | --- | --- | --- | --- | --- | --- | --- | --- | --- |
|  |  | Study 1 | | | | | Study 2 | | | | |
|  | DF | Coef. | SE | HR | *p* | 95% HR CI | Coef. | SE | HR | *p* | 95% HR CI |
| sST2 change | 2 | 0.053 | 0.0172 | 1.054 | 0.002 | 1.019,1.090 | 0.129 | 0.0577 | 1.138 | 0.0253 | 1.016,1.274 |
| logBNP baseline | 2 | 0.53 | 0.2121 | 1.699 | 0.012 | 1.121,2.576 | 0.397 | 0.2083 | 1.487 | 0.0567 | 0.989,2.237 |

sST2 – soluble suppression of tumourigenicity 2; BNP – b-type natriuretic peptide; DF-degrees of freedom; SE-standard error; HR-hazard ratio

**Table S6.** sST2 baseline, follow up, change and percentage on receiver operating characteristics via optimal threshold criteria

|  |  | All Participants | Study 1 | Study 2 |
| --- | --- | --- | --- | --- |
| Baseline | AUC [95% CI] | 0.55 [0.47, 0.64] | 0.45 [0.32, 0.58] | 0.51 [0.38, 0.64] |
|  | Sensitivity | 0.54 | 0.48 | 0.48 |
|  | Specificity | 0.54 | 0.48 | 0.5 |
|  | PPV | 0.21 | 0.11 | 0.33 |
|  | NPV | 0.84 | 0.87 | 0.66 |
|  | Cut-off | 28.1 | 24.1 | 32.8 |
|  |  |  |  |  |
| Follow Up | AUC [95% CI] | 0.62 [0.54, 0.7] | 0.55 [0.43, 0.67] | 0.58 [0.45, 0.7] |
|  | Sensitivity | 0.6 | 0.52 | 0.55 |
|  | Specificity | 0.6 | 0.51 | 0.55 |
|  | PPV | 0.25 | 0.12 | 0.38 |
|  | NPV | 0.87 | 0.89 | 0.71 |
|  | Cut-off | 30.1 | 26.8 | 33.1 |
|  |  |  |  |  |
| Increment | AUC [95% CI] | 0.6 [0.52, 0.68] | 0.62 [0.51, 0.73] | 0.66 [0.55, 0.78] |
|  | Sensitivity | 0.6 | 0.61 | 0.66 |
|  | Specificity | 0.6 | 0.6 | 0.64 |
|  | PPV | 0.25 | 0.17 | 0.48 |
|  | NPV | 0.87 | 0.92 | 0.79 |
|  | Cut-off | 14.7 | 15.6 | 14.2 |
|  |  |  |  |  |
| Change percentage | AUC [95% CI] | 0.59 [0.51, 0.67] | 0.61 [0.5, 0.73] | 0.67 [0.55, 0.79] |
|  | Sensitivity | 0.58 | 0.61 | 0.66 |
|  | Specificity | 0.57 | 0.61 | 0.64 |
|  | PPV | 0.23 | 0.17 | 0.48 |
|  | NPV | 0.86 | 0.92 | 0.79 |
|  | Cut-off | 103.6 | 108.6 | 101.7 |

AUC-area under the curve; PPV-positive predictive value; NPV-negative predictive value

**Table S7.** BNP baseline, follow up, change and percentage on receiver operating characteristics via optimal threshold criteria

|  |  | All Participants | Study 1 | Study 2 |
| --- | --- | --- | --- | --- |
| Baseline | AUC [95% CI] | 0.59 [0.51, 0.67] | 0.65 [0.54, 0.76] | 0.6 [0.48, 0.72] |
|  | Sensitivity | 0.54 | 0.61 | 0.55 |
|  | Specificity | 0.55 | 0.62 | 0.55 |
|  | PPV | 0.21 | 0.18 | 0.38 |
|  | NPV | 0.84 | 0.92 | 0.71 |
|  | Cut-off | 28.4 | 36.1 | 22.1 |
|  |  |  |  |  |
| Follow Up | AUC [95% CI] | 0.58 [0.5, 0.6] | 0.66 [0.55, 0.77] | 0.58 [0.45, 0.7] |
|  | Sensitivity | 0.56 | 0.61 | 0.59 |
|  | Specificity | 0.55 | 0.6 | 0.59 |
|  | PPV | 0.22 | 0.17 | 0.41 |
|  | NPV | 0.85 | 0.92 | 0.74 |
|  | Cut-off | 39.8 | 51 | 29.7 |
|  |  |  |  |  |
| Increment | AUC [95% CI] | 0.48 [0.39, 0.57] | 0.45 [0.32, 0.58] | 0.51 [0.38, 0.64] |
|  | Sensitivity | 0.48 | 0.43 | 0.52 |
|  | Specificity | 0.48 | 0.44 | 0.52 |
|  | PPV | 0.17 | 0.09 | 0.35 |
|  | NPV | 0.8 | 0.85 | 0.68 |
|  | Cut-off | 234.7 | 233.7 | 234.4 |
|  |  |  |  |  |
| Change percentage | AUC [95% CI] | 0.49 [0.4, 0.58] | 0.47 [0.34, 0.6] | 0.5 [0.37, 0.6] |
|  | Sensitivity | 0.48 | 0.43 | 0.52 |
|  | Specificity | 0.48 | 0.44 | 0.52 |
|  | PPV | 0.17 | 0.09 | 0.35 |
|  | NPV | 0.8 | 0.85 | 0.68 |
|  | Cut-off | 118.8 | 103.3 | 113.5 |

AUC-area under the curve; PPV-positive predictive value; NPV-negative predictive value

**Table S8.** sST2 change on receiver operating characteristics via different threshold criteria

|  |  | All Participants | Study 1 | Study 2 |
| --- | --- | --- | --- | --- |
|  | AUC [95% CI] | 0.6 [0.52, 0.68] | 0.62 [0.51, 0.73] | 0.66 [0.55, 0.78] |
| Optimal | Sensitivity | 0.6 | 0.61 | 0.66 |
|  | Specificity | 0.6 | 0.6 | 0.64 |
|  | PPV | 0.25 | 0.17 | 0.48 |
|  | NPV | 0.87 | 0.92 | 0.79 |
|  | Cut-off | 14.7 | 15.6 | 14.2 |
|  |  |  |  |  |
| High Sensitivity | Sensitivity | 0.81 | 0.83 | 0.83 |
|  | Specificity | 0.24 | 0.25 | 0.21 |
|  | PPV | 0.19 | 0.13 | 0.34 |
|  | NPV | 0.85 | 0.91 | 0.71 |
|  | Cut-off | 11.2 | 11.2 | 10.8 |
|  |  |  |  |  |
| High Specificity | Sensitivity | 0.29 | 0.35 | 0.45 |
|  | Specificity | 0.8 | 0.8 | 0.81 |
|  | PPV | 0.25 | 0.19 | 0.54 |
|  | NPV | 0.83 | 0.9 | 0.75 |
|  | Cut-off | 17.5 | 18.5 | 15.2 |

AUC-area under the curve; PPV-positive predictive value; NPV-negative predictive value

**Supplementary Figures**


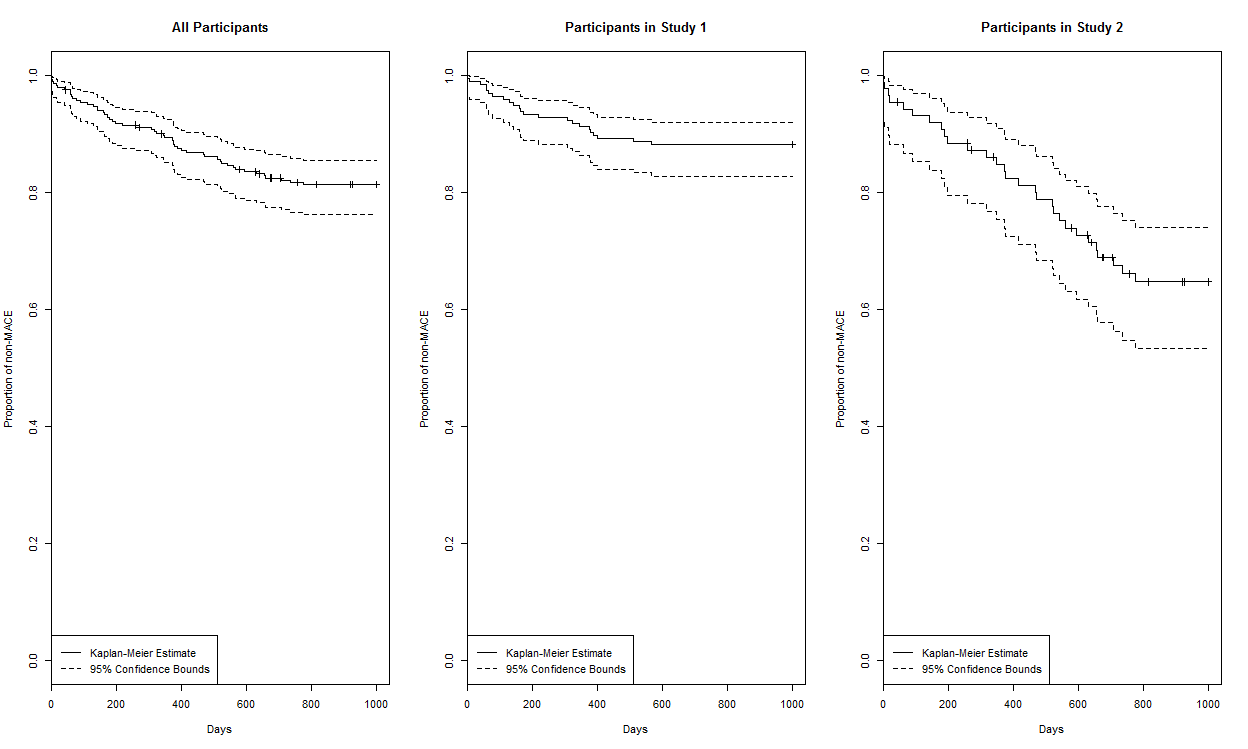


**Figure S1.** Kaplan-Meier survival curves for all participants, study 1 and study 2 being landmarked after follow up.
